# Ambiguous presentation of an intra-abdominal testicular seminoma in a 40-year-old man: a case report

**DOI:** 10.1186/s13256-018-1917-3

**Published:** 2019-01-04

**Authors:** Marius Nkembe Nkembe, Clarence Mbanga Mvalo, Frank Leonel Tianyi, Cisse Demba

**Affiliations:** 1 Franciscan Catholic Health Centre Mayo-Darlé, Mayo-Darlé, Adamawa Region Cameroon; 2 Mankon Sub-divisional Hospital, Mankon, Northwest Region Cameroon; 3 Mayo-Darlé Sub-divisional Hospital, Banyo, Adamawa Region Cameroon; 4 St Elizabeth Catholic General Hospital and Cardiac Centre, Shisong, Northwest Region Cameroon

**Keywords:** Intra-abdominal, Testicular, Seminoma, Case report

## Abstract

**Background:**

Cryptorchidism is the most common congenital malformation of the male genitourinary tract. The cryptorchid testis is most often located in the inguinal canal; however, intra-abdominal locations are not rare. The risk of malignancy in an undescended testis is 10% with the highest risk in an intra-abdominal testis.

**Case presentation:**

Here we describe a case of a 40-year-old fertile man of Fulbe origin who presented with a non-tender lower abdominal mass of 2 months’ duration. A scrotal examination revealed just one testis in the right scrotum, with the contralateral scrotum and inguinal canal being empty. An exploratory laparotomy followed by tumor excision and histopathology revealed a testicular seminoma.

**Conclusion:**

This case report highlights the need for routine scrotal examination in all men presenting with an abdominal mass so as to rule out the possibility of an intra-abdominal seminoma.

## Background

Cryptorchidism is the absence of one or both testes from the scrotum [1]. It is the most common birth defect of the male genitalia [2], with a prevalence of 3% in the full-term male neonate as compared to 30% in preterm neonates [3]. Its prevalence decreases to 1% between the ages of 6 months and 1 year [3].

Several factors have been discovered to predispose to cryptorchidism including prematurity, low birth weight, small for gestational age, twinning, and maternal cigarette smoking and alcohol consumption during pregnancy [4]. If not corrected between the age of 6 months and 1 year, spontaneous descent has been reported to be less likely [5].

The cryptorchid testis predisposes to testicular cancer, ischemia, and infertility later in adulthood [4]. The most common malignant transformation of the undescended testis is testicular seminoma [6].

A review of Medline through PubMed from 15 May 2008 to 15 May 2018 revealed just one case of intra-abdominal testicular seminoma reported in sub-Saharan Africa [7]. Here we present the case of a testicular seminoma in an undescended testis confirmed by histopathology.

## Case presentation

**Fig. 1:**
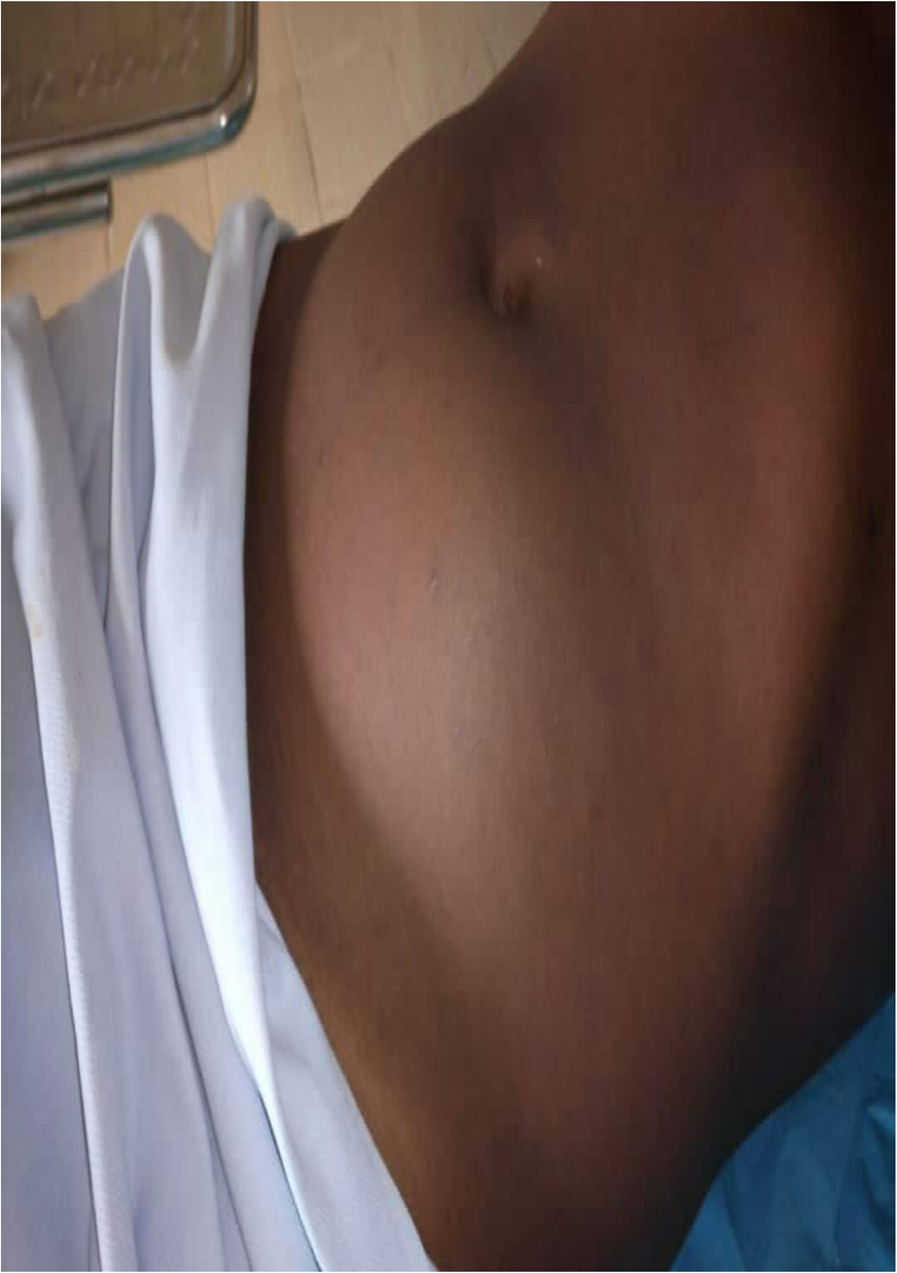
Abdomen of patient showing projection of tumor above pubic symphysis to umbilicus

A 40-year-old Fulbe man from the Adamawa region of Cameroon presented to the out-patient department of our institution with a complaint of a progressively increasing non-tender abdominal mass associated with pollakiuria for approximately 2 months prior to consultation. He is a farmer with no chronic medical condition or past surgeries. He has never been exposed to any carcinogenic substance; he does not consume alcohol, tobacco, or any drugs. He is married and has four children; however, his birth history could not be investigated further. A physical examination revealed a patient who looked well with a blood pressure of 128/82 mmHg, heart rate at 78 beats per minute (bpm), and temperature of 37.4 °C. An abdominal examination revealed a firm, non-tender, non-mobile, hypogastric mass projecting approximately 20 cm above the pubic symphysis (Fig. 1). Examination of his genitalia revealed just one testis in the right scrotum, with the contralateral scrotum and inguinal canal being empty. There were no palpable inguinal lymph nodes or ascites. A neurological assessment revealed conserved muscle forces and sensitivity in all four limbs with all reflexes, particularly the cremasteric and abdominal reflexes, conserved. Paraclinical investigations revealed: no hematuria and proteinuria on urine analysis, normal white cell and platelet count on the full blood count, no blast cells on the blood smear, and a negative human immunodeficiency virus (HIV) serology. A pelvic ultrasound revealed a heterogeneous bean-shaped mass lying above his bladder, approximately 10 cm by 7 cm in size, with five smaller satellite masses. His kidneys, bladder, and bowels had no abnormalities. Given these findings, we had as a probable diagnosis, enlarged mesenteric lymph nodes.

An exploratory laparotomy was scheduled and carried out, with intraoperative findings revealing a highly vascularized mass fixed to the left inguinal ligament, projecting into the retroperitoneum, with several other small satellite masses attached posteriorly. His peripheral bowels, mesenteries, and bladder were all without any visible structural abnormalities.

**Fig. 2:**
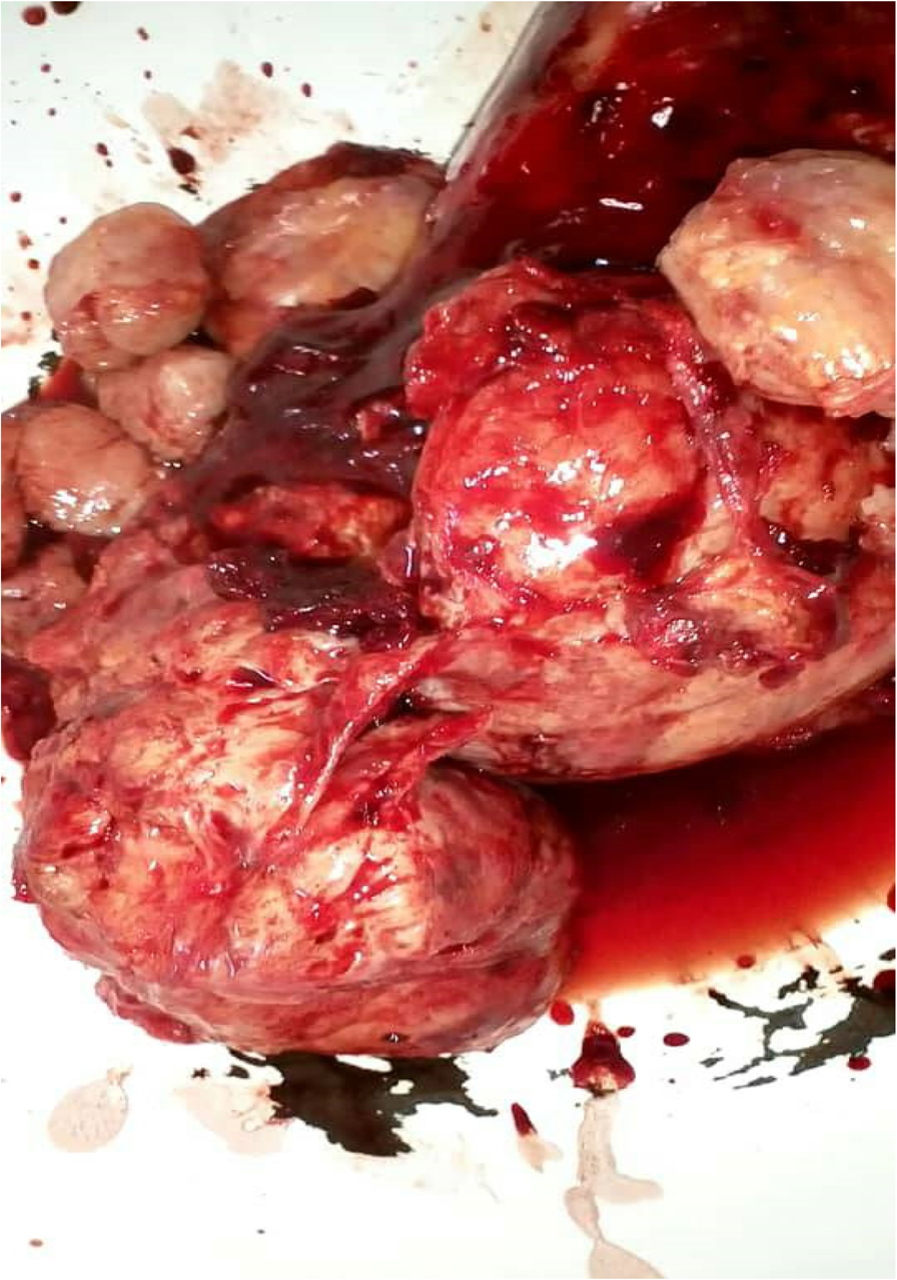
Resected pieces of the tumor

**Fig. 3:**
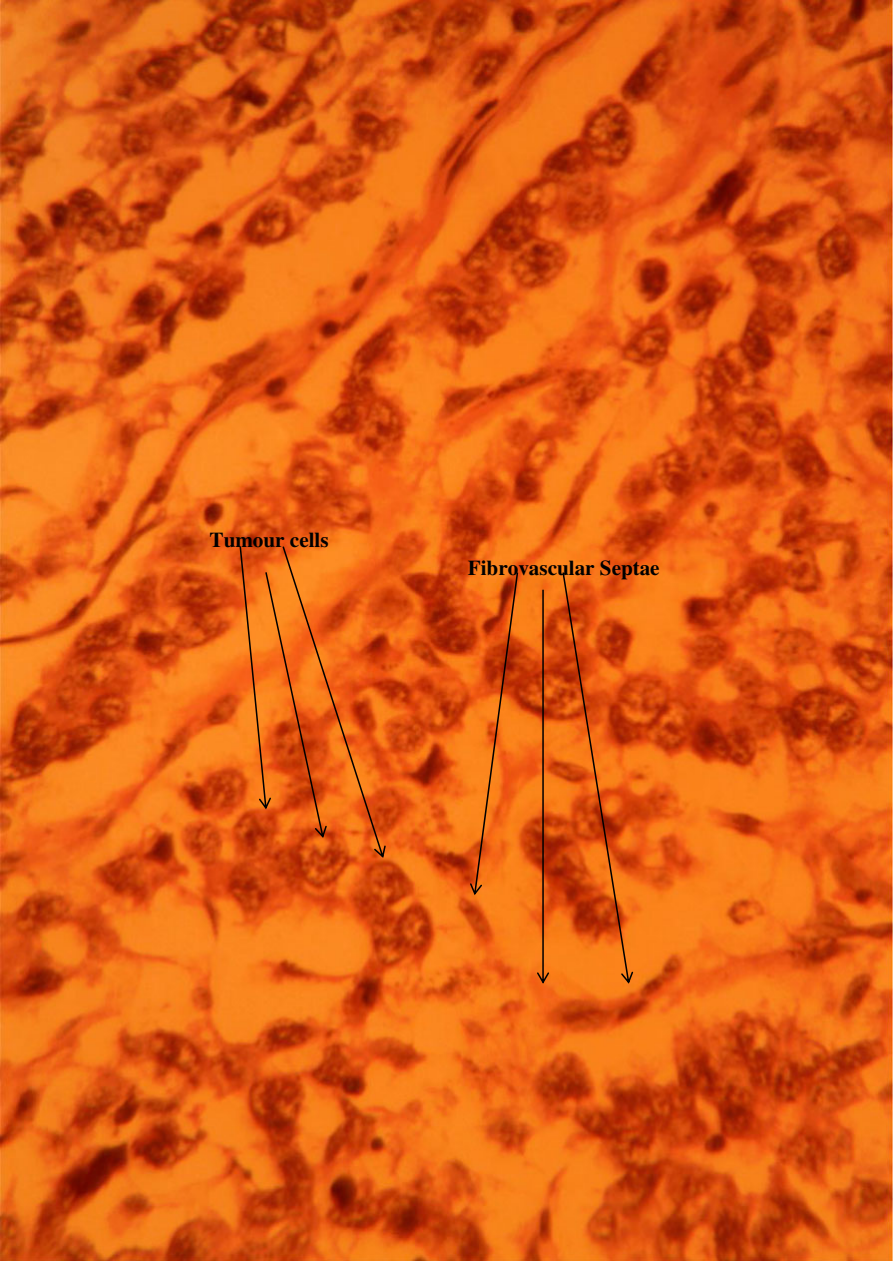
Histopathology slide of sample

Progressive dissection and hemostasis was done to free and resect all the masses. The largest had several lobes attached together, weighed approximately 800 g, and measured approximately 11 cm by 7 cm by 5 cm (Fig. 2). Seven smaller masses were removed with sizes ranging from 3 cm to 6 cm (Fig. 2). Samples of the masses were obtained and sent for histopathology. His postoperative period was unremarkable; he was discharged 7 days after. Histopathology results received 2 weeks later revealed a tumor composed of sheets of fairly uniform polygonal cells having central vesicular nuclei with occasional prominent nucleoli and moderate/abundant brownish/clear cytoplasm. The tumor was divided into lobules by interconnecting thin fibrovascular septa containing a mild, patchy, mixed, inflammatory exudate. All these were suggestive of a seminoma on an undescended testis (Fig. 3).

He was then counselled and referred to see an oncologist for further management. At the time of submission of this manuscript we had not yet received feedback from either our patient or the oncologist.

## Discussion

Testicular seminoma is the most common malignancy in men. We managed a man who had an increasing mass in his lower abdomen with few associated symptoms. The absence of one testis from his scrotum was highly suspicious of a seminoma, despite the fact that he was fertile and had no relevant past history. Given the rarity of reported cases in Cameroon and sub-Saharan Africa as a whole, this case will reinforce already existing literature and permit physicians in our setting to think of a seminoma even in ambiguous presentations such as ours.

Testicular seminoma is a germinal cell tumor of the testicle affecting the germinal epithelium of the seminiferous tubules [8]. It represents approximately half of all testicular germ cell tumors, and is the most common malignancy in males aged 15 to 35 years [8, 9]. Patients with a history of cryptorchidism are 10 to 40 times more likely to develop testicular seminomas [10]. Our patient admitted to feeling just one testicle from childhood, hence the undescended testis is most probably the cause of the seminoma.

Seminoma is a pathology diagnosis. Patients usually present with a painless testicular lump; however, an intra-abdominal testicular tumor manifests with signs of an increasing abdominal mass sometimes associated with signs of partial bowel obstruction, bladder compression, or even pain from torsion [11]. The increase in size of the mass progressively causes bladder compression resulting in difficulties maintaining a full bladder hence pollakiuria as seen in our case.

Imaging techniques such as ultrasonography, computed tomography scan, and magnetic resonance imaging usually show a well-defined heterogeneous retroperitoneal mass with no ischemia or calcification; findings which are nonspecific and could mimic other frequent pathologies such as enlarged lymph nodes and sarcoma [12]. The definitive diagnosis is therefore done by histopathology. Histopathological findings typically consist of sheets of relatively uniform tumor cells with abundant clear/watery cytoplasm having large central nuclei with prominent nucleoli [13]. These cells are usually divided into poorly demarcated lobules by delicate fibrous septa and have minimal mitotic figures. The histopathological findings of our sample were compatible with those reported in the literature.

Although a malignant neoplasm, testicular seminoma is one of the most curable cancers with a survival rate above 95% if discovered early [14]. Surgical resection of the tumor followed by pathology studies is recommended in most cases [14]. This is to prevent tumor rupture, torsion, and complications of peripheral organ compression. Once diagnosis is certain, other investigations including a chest X-ray, abdominal computed tomography scan, beta-human chorionic gonadotropin levels, and alfa-fetoprotein levels are done to facilitate staging and orientate further management (chemotherapy or radiotherapy) [15]. Given our resource-limited setting, we decided to send our patient for proper investigation and management by an oncologist.

## Conclusions

Testicular seminoma is a frequent malignancy in middle-aged men and cryptorchidism further predisposes to its onset. We therefore reiterate the need for a scrotal examination in men presenting with an abdominal mass, so as to rule out an intra-abdominal seminoma.
